# pH-Responsive Mesoporous Silica and Carbon Nanoparticles for Drug Delivery

**DOI:** 10.3390/bioengineering4010003

**Published:** 2017-01-18

**Authors:** Miguel Gisbert-Garzarán, Miguel Manzano, María Vallet-Regí

**Affiliations:** 1 Departamento de Química Inorgánica y Bioinorgánica, Facultad de Farmacia, Universidad Complutense de Madrid, Instituto de Investigación Sanitaria Hospital 12 de Octubre i+12, Plaza Ramón y Cajal s/n, E-28040 Madrid, Spain; migisber@ucm.es (M.G.-G.); mmanzano@ucm.es (M.M.); 2 Networking Research Center on Bioengineering, Biomaterials and Nanomedicine (CIBER-BBN), Madrid 28029, Spain

**Keywords:** mesoporous nanoparticles, pH-responsive, cancer, stimuli-responsive

## Abstract

The application of nanotechnology to medicine constitutes a major field of research nowadays. In particular, the use of mesoporous silica and carbon nanoparticles has attracted the attention of numerous researchers due to their unique properties, especially when applied to cancer treatment. Many strategies based on stimuli-responsive nanocarriers have been developed to control the drug release and avoid premature release. Here, we focus on the use of the subtle changes of pH between healthy and diseased areas along the body to trigger the release of the cargo. In this review, different approximations of pH-responsive systems are considered: those based on the use of the host-guest interactions between the nanocarriers and the drugs, those based on the hydrolysis of acid-labile bonds and those based on supramolecular structures acting as pore capping agents.

## 1. Introduction

The application of nanotechnology in health and medicine, so-called nanomedicine, is one of the most promising and exciting research areas nowadays. The design of nanoparticles to address disease and to monitor and protect human health is expected to revolutionize the medical field in the next few decades. In this sense, the capacity of producing nanoparticles in the range of 20–200 nm in diameter has fueled the design of materials large enough to escape clearance through the kidney and small enough to present long circulation times into the bloodstream.

Although nanoparticles have been applied against lung [1], kidney [2], rheumatoid arthritis [3], diabetes [4] or neurodegenerative diseases [5], among others, the first steps of nanomedicine research have been mainly focused on the challenging area of cancer, either on diagnosis or treatment.

Current cancer treatments concern the use of radiotherapy, surgery or chemotherapy. Unfortunately, chemotherapeutics are administered systemically and do not show selectivity for cancer cells. Because of that, chemotherapy is considered to be aggressive for patients, since the drugs distribute all along the body instead of only near the damaged area. Then, it would be desirable to target the chemotherapeutics only to cancer cells. A smart approximation would be the use of nano-sized therapeutics for cancer therapy since: (1) they can retain the drugs until reaching the tumor, thus not affecting the healthy tissues; (2) the therapeutic efficacy of poor water-soluble drugs might be enhanced by incorporating them into the nanocarriers; (3) nanoparticles can protect the drugs from any possible degradation on their way to their therapeutic target; (4) it is possible to introduce two or more drugs in the nanocarriers, so it is possible to design combined therapies; and (5) nanoparticles can be decorated with targeting moieties capable of recognizing concrete cancer cells.

An ideal nanoparticle for drug delivery should be able to entrap the maximum amount possible of pharmaceutical agents. Then, those nanoparticles should be intravenously injected into the bloodstream, carry the drugs to the tumor and, finally, release the drugs only there. However, reaching the tumor without being removed from the bloodstream is still challenging for the nanoparticles. To avoid that, the surface of the nanoparticles can be decorated with hydrophilic layers to prevent opsonization (and therefore, the action of macrophages) [6,7,8]. The nanoparticles can accumulate in the tumor via passive targeting due to the abnormal vasculature of solid tumors (the so-called Enhanced Permeability and Retention effect) [9] and then selectively internalize the cancer cells via active targeting [10].

### 1.1. Types of Nanoparticles

#### 1.1.1. Non-Porous

In the last few years, a number of non-porous nanoparticles for biomedical applications have been developed. It is possible to find both organic or inorganic platforms, such as liposomes, polymeric nanoparticles, dendrimers, magnetic nanoparticles or metal nanoparticles, among others [11]. In fact, there are some examples of those non-porous platforms commercially available, such as: (1) paramagnetic iron oxide nanoparticles (e.g., Venofer, Resovist); (2) liposomes (e.g., Doxil, Caelyx); (3) biodegradable polymer nanoparticles (e.g., Somavert, Cimzia); or even (4) drug-antibodies conjugates, in which those antibodies guide the drug towards the targeted cells (e.g., Mylotarg) [12].

#### 1.1.2. Mesoporous Nanoparticles

Mesoporous nanoparticles have recently attracted the attention of nanomedicine researchers thanks to their robustness, their high loading capacity and the easy chemical functionalization of their surface. This offers a great potential for targeted drug delivery and controlled release of chemotherapeutic drugs.

##### Mesoporous Silica Nanoparticles 

**Figure 1:**
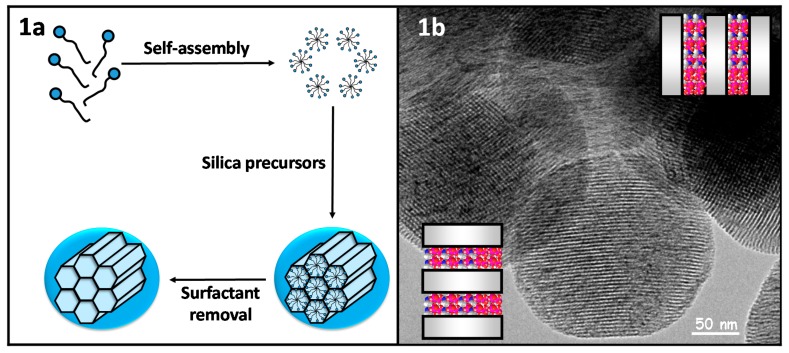
(**a**) Scheme synthesis of mesoporous silica nanoparticles through a modified Stöber; (**b**) Transmission Electron Microscopy micrography of the pore distribution of MCM-41-type mesoporous silica nanoparticles.

The synthesis of mesoporous silica nanoparticles (MSNs) is based on the sol-gel process, in which successive hydrolysis and condensation steps of the silica precursors lead to the formation of a network of silica. That polymerization process is carried out in the presence of surfactants as structure-directing agents (Figure 1a). The final mesostructure would depend on the type and concentration of surfactants and also on certain experimental parameters, such as temperature. Then, removing the surfactant would lead to mesoporous materials with empty mesopores, where drug molecules would be loaded. A modification of the Stöber method is employed to obtain nanoparticles with mesoporous structures, which is based on using very dilute conditions during the sol-gel process. In a typical synthesis, cetyltrimethylammonium bromide (surfactant) is dissolved in pure water in the presence of sodium hydroxide so that the surfactant organizes in cylindrical micelles. After that, tetraethyl orthosilicate (silica precursor) is added dropwise, and the silica network is formed through the hydrolysis and condensation of the silica precursor around the micelles. Finally, the surfactant is removed from the mesopores through extraction with either a methanol/HCl or ethanol/ammonium nitrate solution, giving rise to nanoparticles with a hexagonal distribution of empty pores (Figure 1b).

##### Mesoporous Carbon Nanoparticles 

In the last few years, mesoporous carbon nanoparticles have received increasing attention by nanomedicine researchers since mesoporous carbon nanoparticles (MCNs) show similar structural properties as MSNs. Moreover, MCNs show enhanced laser absorption in the near-infrared (NIR) region that leads to an efficient conversion of light into heat that can be applied to antitumor therapy [13]. The synthesis of MCNs can be accomplished by either hard or soft templating approaches [14]. The hard templating approach is based on the use of presynthesized organic or inorganic templates. The template serves as a mold for the replication of the mesoporous carbon materials, and no significant interactions between the carbon precursors and the template are observed. A typical synthesis involves four steps: (1) preparation of the porous silica template; (2) impregnation of the silica template with the precursors; (3) cross-linking and carbonization of the organic precursors (e.g., phenolic resins, sucrose); and (4) dissolution of the silica template. On the other hand, the soft templating approach is based on the generation of nanostructures through the self-assembly of organic molecules, such as surfactants.

### 1.2. Stimuli-Responsive Nanoparticles

**Figure 2:**
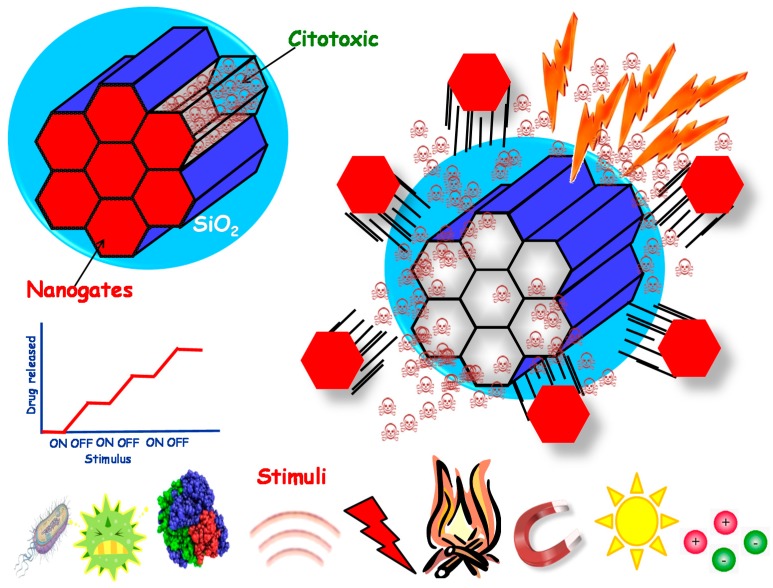
Stimuli-responsive nanoparticles where the drug release can be triggered through the application of many different stimuli.

As has been mentioned before, nanoparticles are attractive in drug delivery applications due to their ability to host drugs within them. An efficient drug delivery system would travel along the body without releasing the cargo until reaching the diseased area, where the drugs would be released. To achieve that, the concept of stimuli-responsive drug delivery arises. It is based on the use of stimuli to trigger the release of the payload only under specific conditions, i.e., those of the damaged area. This concept is particularly useful when using mesoporous silica or carbon-based nanoparticles because they present an open structure. Then, the drugs can be loaded through diffusion within them, but the cargo can also diffuse out when placed in aqueous media. This clashes with the desired release of the drugs only in the target area. Although the electrostatic interactions between nanocarriers and guest molecules may help to retain the cargo, as will be seen in the following section, an extended strategy is the use of molecular structures capable of blocking the mesopores. These structures are referred to as gatekeepers, which are organic or inorganic structures capable of hampering the release at physiological conditions and suffering some kind of structural change when a certain stimulus is applied, which would open the pores and trigger the drug release. The gates can be opened through external or internal stimuli (Figure 2).

#### 1.2.1. External Stimuli-Responsive Nanocarriers

External stimuli-responsive nanocarriers are those in which the opening of the pores is mediated by some stimulus coming from the outside of the body. The main advantage of this kind of system is that the stimulus can be modulated, opening and closing the gates on-demand in many cases, as they are applied through external equipment. The literature provides examples of temperature [15,16], magnetic [17,18], light [19,20,21] or ultrasound-sensitive [22] systems. The use of magnetic and thermo-responsive nanoparticles has also been reported, as they are synergic stimuli because heat can be generated by applying an alternating magnetic field [23,24].

#### 1.2.2. Internal Stimuli-Responsive Nanocarriers

Internal stimuli-responsive nanocarriers are those in which the behavior of the gate is modulated by some stimulus from the inside of the body. It is well-known that during some diseases, the determined enzymes may be overexpressed [25] or the concentration of reducing agents may increase inside the cell with respect to the outside [26]. These abnormal situations lead to enzyme- [27,28] and redox-responsive [29,30] drug delivery systems, among others.

Interestingly, as will be discussed during the following sections, the value of the pH is not kept constant along the body. Then, these subtle changes in pH can be taken advantage of to design pH-responsive drug delivery systems that only release the payload when the pH deviates from the physiological value.

#### 1.2.3. The Role of pH in Drug Delivery

The maintenance of the different physiological pHs along the body is of vital importance for the organism to keep alive. For instance, variations in pH of body fluids within 7.35 and 7.45 can be corrected, but greater alterations may lead to acidosis or alkalosis events that may even cause death. However, that rule does not always apply, and these variations can be taken advantage of in different scenarios throughout the body to design pH-responsive nanocarriers.

##### Digestive System

It is well known that the pH is not kept constant along the organs of the gastrointestinal tract due to the different conditions in which the nutrients are digested. This fact has been exploited for the design of oral drug delivery therapeutic carriers [31,32]. In the stomach, the pH is as low as 1–2, and it is progressively neutralized until reaching pH 7–8 in the colon and rectum. The pH in the stomach is lowered by the secretion of protons by the parietal cells, which can be suppressed by different hormones. As a protective mechanism, the epithelium secrets bicarbonate ions to regulate the pH. After leaving the stomach, the bile salts are responsible for increasing the pH until reaching the colon [33].

##### Extracellular Tumor Microenvironment

One general feature of inflammatory processes and solid tumor tissues is the presence of extracellular acidity, with a pH lower than the expected one in healthy tissue. It is well established that cells need adenosine triphosphate (ATP) to carry out their physiological functions. ATP can be obtained from glucose through different pathways. In the presence of oxygen, non-tumor cells obtain the ATP through the Krebs cycle, in which glucose is first transformed to pyruvate, which then is mainly oxidized to ATP via oxidative phosphorylation. The rest of the pyruvate is converted into lactate through anaerobic glycolysis. However, it was observed that cancer cells tend to convert almost all glucose into lactate independent of the presence of oxygen, which, surprisingly, was also observed in non-tumor cells, although to a lesser extent. This production of lactate is known as the “Warburg effect” [34]. The Warburg effect is the main effect responsible for the acidification of the extracellular tumor environment, although the pentose phosphate pathway also contributes thanks to the hydration of CO_2_ catalyzed by carbonic anhydrase. Lactate plays an important role regarding cancer cells, such as: contributing to their immune escape, promoting chronic inflammation in tumor environments, enhancing the motility of the tumor or contributing to angiogenesis, among others. The acidic extracellular pH is also necessary for some enzymes to be secreted by cancer cells, such as cathepsin B and cathepsin L [35]. This acidification of the extracellular tumor environment has been quantified for several cancer cells, and it has been determined to be 6.5–7 [36,37,38].

##### Intracellular pH

The differences in pH among the different cellular organelles and compartments has been taken advantage of to design a number of pH-responsive systems. Interestingly, although there exist differences between the extracellular pH of tumor cells and that of non-tumor cells, the intracellular pHs of both kinds of cells have been observed to be the same. Substances can be internalized by cells through the endocytic pathway. It comprises endosomes and lysosomes, and the pH becomes more acidic as the substances go from the endocytic vesicles (pH 6.5) to the lysosomes, where the pH has been reported to be as low as 4.5. The different organelles have an intrinsic capacity to buffer changes within them slightly. This buffering capacity is provided by weak acids and bases present there. However, this buffer is finite, and therefore, there must be other mechanism to control the pH of each compartment. That precise regulation of the pH is provided by the presence of proton-pumping ATPases. The injection of protons by these pumps together with the existence of channels capable of introduce negative ions gives rise to the final pH of each organelle [39]. Then, this drop in the pH can be taken as an advantage to design intracellular-responsive nanocarriers that are supposed to release the cargo only inside the cell, as will be detailed throughout this review.

## 2. Host-Guest Interactions

**Figure 3:**
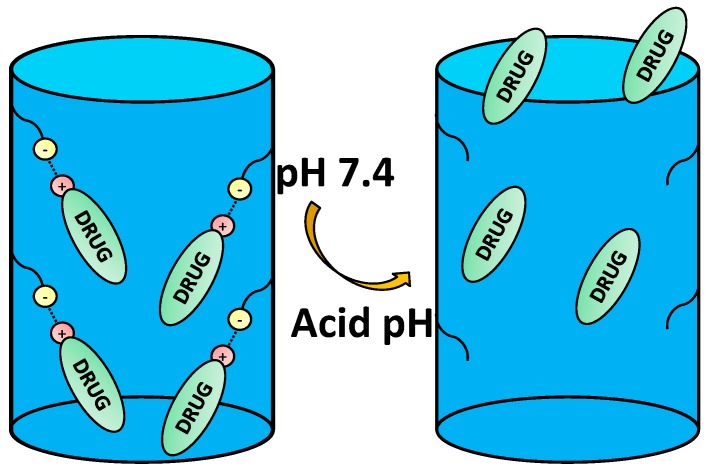
Example of host-guest interaction-based drug delivery involving cationic drugs.

An extended strategy to provide the nanocarriers with pH sensitivity is through the electrostatic interaction between the functional groups from the carrier and those of the drugs at physiological pH, as well as the lack of them when pH varies (Figure 3).

Depending on the ionic nature of the therapeutic agents, a specific nanocarrier functionalization should be chosen. Generally speaking, positively-charged nanocarriers should be used when using an anionic drug in order to maximize the loading capacity and achieve a controlled released and vice versa [40,41,42].

One of the most used drugs against cancer is doxorubicin (DOX). DOX is a cationic drug that has been widely used for more than 30 years to treat different types of tumors. It has shown great treatment potential, but its lack of cancer cell selectivity makes it a double-edged sword because it kills not only malignant, but also healthy cells. The action mechanism is complex, but, briefly, it enters the cell through diffusion due to its high affinity to bind to the proteasome of the cytoplasm. Once there, it interferes with the topoisomerase II-DNA complex, leading to the formation of double-stranded breaks of the DNA, causing cell death [43].

Although the literature on mesoporous nanoparticles mostly provides DOX-based papers, the study of the host-guest interactions with some other drugs, such as ibuprofen, 5-fluoracil, mitoxantrone, methotrexate or sulfasalazine, has also been reported [31,44,45,46].

### 2.1. DOX Loading and Release Mechanism

DOX can be retained through different mechanisms. When using MSNs, the storage takes place through the electrostatic interaction between DOX and the silanol groups of the surface of the nanoparticles. It is worth noting that the pKa value of the amino group of DOX is 8.2 [47] and that of silanol groups of MSNs is 3.5 [48], so at pH 7.4, DOX is partially positively charged, and the silanol groups are deprotonated, thus both remaining electrostatically attracted. However, when the pH drops below this value, silanol groups become partially protonated, as well as DOX amino groups; therefore repulsion interactions appear, thus leading to the release of DOX. It is possible to tune the drug adsorption and release through the introduction of silylated organic groups in a process known as functionalization [49].

When MCNs are used, the storage takes places thanks to the π-π staking interactions between the aromatic rings of DOX and the aromatic carbonaceous structure of the nanocarriers. They are non-covalent attractive interactions between aromatic molecules due to the sp^2^ hybridization that can be disrupted when the pH drops, thus allowing the release of the drug [50].

It is possible to go further and combine these two different types of storage interactions by synthesizing mesoporous nanoparticles that combine both silica and carbon moieties [51]. Hybrid core-shell magnetic mesoporous nanoparticles with silica or carbon shell have also been reported [52,53,54], thus providing nanocarriers with not only the above-mentioned storage properties of MSNs and MCNs, but also promising applications in hyperthermia, photothermal therapy or imaging.

Both the drug loading and release take place through a diffusion process [55]. In particular, the total amount of DOX loaded into the silica nanocarriers will depend on both the pH of the loading DOX solution and the kind of functionalization of the nanocarriers. Loading in basic media is an extended strategy because repulsion interactions due to protonation processes are lowered, and DOX solubility is decreased [56,57]. In contrast, when the loading process is carried out in acidic media, the diffusion is slowed down by the repulsion forces, thus leading to a lower drug storage capacity and, therefore, lower therapeutic efficacy. Nanocarrier functionalization is closely related to this. Functionalization with positively-charged functional groups will lead to repulsion forces. On the other hand, functionalization with negatively-charged functional groups will provide higher loading capacity because of the deprotonated state of these functional groups in basic media.

### 2.2. Host-Guest Interaction-Based Nanocarriers

Although the host-guest interactions are supposed to retain the drugs inside the nanocarriers until the pH stimulus is applied, what is actually observed is that even at pH 7.4, a small release of DOX takes place despite the fact that repulsion interactions at this pH are supposed to be small [58]. Then, it is easy to find examples of nanocarriers that combine the host-guest interaction as the pH-responsive mechanism with other stimuli-responsive moieties capable of blocking the pores.

#### 2.2.1. Dual Stimuli-Responsive Nanocarriers

It is usual to combine more than one stimulus in a nanocarrier, such as pH and light, pH and redox or pH and temperature, among others, which leads to nanoparticles capped with multiresponsive nanogates. Responsive gates that complement the pH responsiveness of DOX will be described here, while the others will be discussed later. Although pH- and enzyme-responsive systems have been reported [59], the most abundant are those that are pH and redox responsive. This approximation only allows the opening of the pores when a certain concentration of glutathione (GSH) is present in the medium, and then, thanks to the lower tumor pH, DOX is released in a controlled manner. GSH is the most abundant non-protein thiol in mammalian cells and acts as a reducing agent that maintains enzymes in an active state. In cancer cells, the concentration of GSH is higher than that of normal cells, which in fact seems to promote multidrug and radiation resistance, but also provides a good tool to trigger the release of drugs [60]. The disulfide bond is a widely-used redox-labile bond, which can be used to coat MSNs with lipid layers through them being capable of retaining DOX at neutral pH and being cleaved after exposure to high GSH levels, showing high efficacy in vitro with MCF-7 cells [61]. GSH can also dissolve manganese silicate nanoparticles that can act as both gatekeepers and Mn (II) paramagnetic center sources for magnetic resonance imaging (MRI) [62].

#### 2.2.2. Nanocarriers for Dual Therapy

An extended strategy is the use of combined therapies, such as chemo- and photothermal therapy (PTT), as they have been shown to be synergistic. It is usual to cap the mesopores with photothermal agents, such gold nanorods [63], CuS nanoparticles [64,65] or carbon-based moieties [66,67,68]. Gold nanorods have shown high efficiency in vivo in combination with DOX due to their good biocompatibility and their surface plasmon resonance [63], while the small CuS p-type semiconductor nanoparticles make PTT possible due to NIR light absorption derived from energy band transitions, achieving high efficacy in combined therapy in vitro [65]. Graphitic carbon is capable of absorbing NIR light as well and has been used to design core-shell nanoparticles that also take advantage of π-π stacking between DOX and carbon walls, showing high efficiency to kill SK-BR-3 cells in vitro when combined with DOX [66].

## 3. Acid-Labile Bonds

Labile bonds are widely used in drug delivery as they are supposed to be stable until some stimulus is applied. Although there exist external stimulus labile bonds, such as light-labile bonds [21], the use of internal stimuli, such as redox [69], enzyme [28] or pH, is more usual, which will be discussed in further detail in the following section, due to the abnormal levels of reductive species, enzymes or pH value in cancer environments.

Acid-labile bonds have been widely used in the design of a number of biocompatible mesoporous drug delivery systems. Some of them are shown in Table 1.

### 3.1. Hydrazone Bond

The pH-sensitive hydrazone bond has been widely used in the design of prodrugs as it is known to be stable at physiological pH and suffer fast hydrolysis at pH 5 and also because of the low complexity of its chemistry [102]. The term prodrug refers to masked forms of active drugs that are supposed to be inactive in the physiological medium until some stimulus is applied, after which the prodrug suffers some kind of conformational change that reconverts it into the original molecule with biological activity, thus only allowing the release in the desired area, increasing the targeting ability of the system [103].

**Figure 4:**
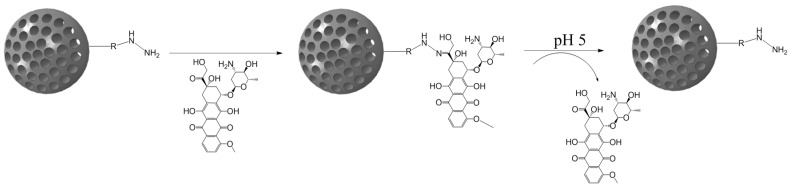
Mesoporous silica nanoparticle (MSN)-doxorubicin (DOX) via hydrazone bond on the surface. The acid-labile bond is cleaved at acidic pH, and the drug is released only in the desired area.

Although cisplatin-based systems have been reported [70], the most extended procedure is the complexation of DOX to the nanocarrier via its ketone group to form the hydrazone bond (Figure 4).

DOX can be conjugated to the external surface of the carrier [71] or to the inner pore surface [72]. This last approximation provides a more sustained release because DOX is retained by both the hydrazone bond and the π-π stacking interactions with the covalently-linked aromatic DOX molecules [73].

As previously mentioned, physically-adsorbed DOX diffuses freely in acid medium. To avoid that, biocompatible gold nanoparticles (AuNPs) have been used as capping agents through the acid-labile hydrazone bond. The AuNPs would block the pores at physiological pH while allowing the drug release in cancerous environments. This approach has been used in core-shell magnetic nanoparticles capable of developing hyperthermia and chemotherapy with success in in vitro studies with HeLa cells [74] and to prevent the premature release of ^19^F loaded in MSN for MRI purposes [75]. Here, AuNPs have been only used as pore blocking agents, but it is worth noting that because of their surface plasmon resonance, they could also be used to carry out PTT [76]. The anchoring of hyaluronic acid as both the targeting agent and gatekeeper to MSNs through the hydrazone bond has also been reported [77].

### 3.2. Acetal Bond

A component of bee venom with promising applications in cancer treatment, melittin, has also been reported as a prodrug, in this case through an acetal bond [78]. This pH-responsive bond has also been used as linker between MSNs and small nanoparticles acting as capping agents (AuNPs [79], graphene quantum dots [80] or lanthanide-doped ultra-small upconverting nanoparticles [104]). The term quantum dot refers to a kind of semiconductor nanocrystal with unique electro-optical properties that depend on both the material and the dimensionality, which find in imaging a great field of application [105]. On the other hand, upconversion is an optical process through which some materials are capable of converting low-energy photons into high-energy photons, thus also establishing these nanoparticles as promising candidates in the field of imaging [106]. The use of biocompatible polymers to prevent premature drug release is very common in this field. Polymers can both act as passive or active gatekeepers. When used in a passive way, polymers can be linked via pH-sensitive bonds, such as the acetal bond [81,82], and their function is mainly blocking the pores. On the other hand, active polymers also block the pores, but the release takes place when they suffer some kind of conformational changes under certain stimuli. This topic will be discussed in the following sections.

### 3.3. Imine-Based Bond

**Figure 5:**
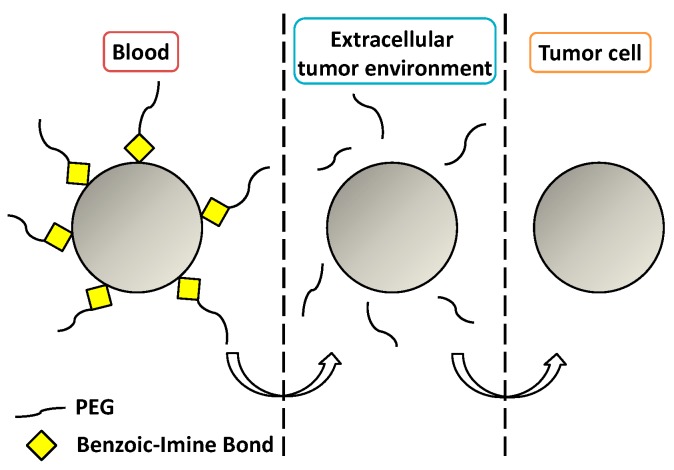
Cleavage of a benzoic-imine bond to overcome the PEG dilemma.

Imine bonds are condensation products of primary amines with carbonyl compounds. They are widely used because although they suffer fast hydrolysis at a pH near five [83]; they also show a good rate of hydrolysis at pH 6.8 (i.e., extracellular tumor pH). In particular, benzoic-imine bonds are interesting because they might help to overcome the so-called PEG dilemma. It is possible to PEGylate the nanocarriers via imine chemistry, enhancing the circulation time and cleaving the PEG shell once the nanocarrier has been accumulated in the tumor due to the EPR effect, leading to positively-charged nanoparticles that show better internalization [84,85] (Figure 5).

Imine-bond-based compounds are also known as Schiff bases. They find application in a wide range of biomedical processes, from antimicrobial, analgesic or antitubercular activity [107], to amine masking agent in the design of prodrugs [108]. Schiff bases are also useful as cross-linking agents. For instance, it is possible to design pH-sensitive protective layers onto the nanocarrier by reacting amine-containing with carbonyl-containing macromolecules, such as chitosan with dialdehyde starch [86], dextrin with tetraethylenepentamine [87] or glutaraldehyde with polyethyleneimine [88]. Schiff bases have also been used to design a hyper-branded polyglycerol layer to provide MSNs with pH-sensitivity [89].

### 3.4. Ester-Based Bond

Although polymers linked through an ester bond or even polymers based themselves on ester bonds, such as poly(β-amino ester), have been reported as capping agents [90,91], this pH-sensitive bond finds further application as a linker between nanocarriers and more complex pore-blocking structures. As boronate ester hydrolysis is known to be reversible [109], researchers have taken advantage of that designing nanocarriers capped with small Au [92] or Fe_3_O_4_ [93] nanoparticles through a boronate ester bond as on-off release systems. On the other hand, this pH-sensitive bond has been used to functionalize nanocarriers with lactobionic acid, which not only is a targeting agent for the galactose receptors of HepG2 cells [94], but also serves as an intermediate in the design of nanocarriers capped with macromolecules, such as bovine serum albumin [95], chitosan [96] or covalently-linked β-cyclodextrin with applications in imaging [97] and dual pH and sugar responsiveness systems [98,99].

### 3.5. Coordination Bond

To the best of our knowledge, Shunai Che and coworkers were the first who reported coordination bonding-based mesoporous silica nanoparticles [110] and laid the foundations of this pH-responsive strategy. They are based on the host-metal-guest coordination bond, so that when the pH drops, either the host-metal or the metal-guest may be cleaved. Although MSNs with ultra-small iron oxide nanoparticles inside them as MRI and coordinating agents have been reported [111], nanoparticles are usually functionalized with amino-based groups that can get coordinated to metal ions, such as Zn^2+^ [112,113], Fe^3+^ [114], Co^+2^ [115], In^3+^ [116] or Cu^2+^ [117,118], among others. Depending on which of the last interactions is stronger, it is possible to simply deliver a drug or to deliver both drugs and ions, which are known to have therapeutic properties [119]. As pH drops, proton concentration increases, which leads to competitive bonding processes between guest molecules, protons and metal ions, because both metal ions and protons are Lewis acids, which compete to combine with ligands, which are Lewis bases [120,121]. This pH-sensitive bond has also been used to design dual responsive nanocarriers, such as pH and redox [122] or pH and photo responsive [123]. Taking advantage of the host-guest interactions between DOX and nanocarriers, it is possible to achieve dual releasing by coordinating other drugs with metal ions [124], thus leading to the release of both drugs controlled, on the one hand, by the host-guest interactions and, on the other hand, by the nature of the interactions of the coordination bond as pH drops.

## 4. Supramolecular Structures as Pore-Capping Agents

### 4.1. Disassembling Gatekeepers

#### 4.1.1. Self-Immolative Polymers

**Figure 6:**
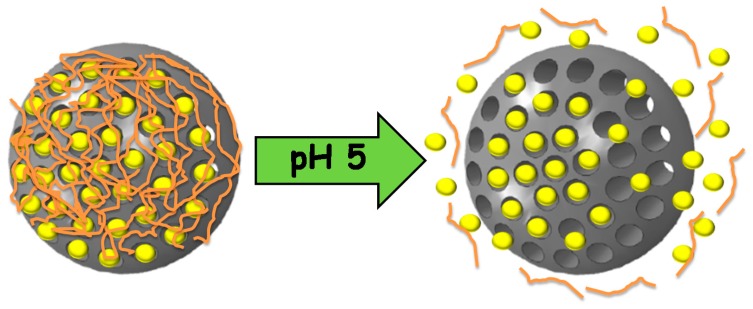
Mesoporous silica nanoparticles capped with self-immolative polymers that disassemble at acidic pH, triggering the release of the cargo.

We recently reported for the first time the use of self-immolative polymers as gatekeepers in MSNs [125]. These polymers present one or more triggering units in the backbone that, after a certain stimulus is applied, start the progressive degradation (self-immolation) of the polymer [126]. In our case, a linear polyurethane with a pH-responsive moiety at the end of the backbone is used. At physiological pH, the BOC triggering unit avoids the degradation of the polymer, while at pH 5, it is cleaved. Then, the self-immolation starts, leading to a progressive opening of the pores and drug release (Figure 6).

#### 4.1.2. Small Nanoparticles

In Section 3, a number of systems based on small nanoparticles linked through acid-sensitive bonds as gatekeepers has been shown. However, the use of nanoparticles capable of directly disassembling as pore-capping agents has also been reported in the literature. ZnO quantum dots were first reported as disassembling gatekeepers in MSNs. This inorganic structure is known to be stable at physiological pH, but it rapidly dissolves at pH lower than five, i.e., inside the cells, thus allowing the drug release. Moreover, ZnO quantum dots are not cytotoxic unless they dissolve. In that case, the resultant Zn^2+^ ions have been demonstrated to damage the DNA through the generation of radical oxygen species [127]. This is the reason why this kind of nanocarrier can induce high cytotoxicity even at low concentrations [128]. This inorganic gatekeeper has been used to design dual stimuli [129], as well as dual drug release [130,131] and enhanced endosomal escape nanocarriers [101].

There are also systems based on pH-responsive hydroxyapatite nanoparticles. As these nanoparticles degrade when pH drops, they only allow the release in tumoral tissues and have the advantage of being biocompatible, even after dissolving [132,133]. Calcium phosphates and carbonates have also been reported as gatekeepers by deposition of inorganic layers on MSNs, as this provides efficient capping with high cytotoxicity [134,135,136,137]. MSNs capped with small MnO inorganic nanoparticles have also been reported, which not only act as gatekeepers that dissolve when the pH drops, but also provide an easy way to carry out MRI [138,139].

### 4.2. Pore Capping through Electrostatic Interactions

An efficient strategy to block the pores is the use of non-covalently-bonded structures that can prevent the drug release at neutral pH and allow it at a lower pH due to the reduction of the electrostatic interaction between the nanocarrier and the gatekeeper or even within the gatekeeper itself. Table 2 summarizes some of the most used polyelectrolytes in the design of pH-responsive nanocarriers.

#### 4.2.1. Polyelectrolyte Multilayers

Although other approaches have been reported [57], the most extended procedure to synthesize these gatekeepers is the layer-by-layer self-assembly technique. Its main advantage is that it allows a precise control of the layer thickness and the molecular organization of the layers [151], as well as swelling behaviors and tunable permeability and elasticity [152]. This smart gate is based on the electrostatic interactions between cationic and anionic polyelectrolytes. At neutral pH, the layers are close to each other, leading to a robust coating that prevents premature release. However, when the pH drops, the interaction weakens, and the multilayer disassembles, thus allowing the release. Chitosan has attracted the attention of a number of researchers as a polycation because its amine group becomes protonated under mildly acidic conditions [56,151]. The use of poly(allylamine hydrochloride) (PAH) as a polycation is also very extended, especially in combination with poly(styrene sulfonate) (PSS) as a polyanion, although other polyanions have been used [144,145], due to its unique pH sensitivity and biocompatibility [146].

#### 4.2.2. Electrostatic Interactions between the Surface and the Gatekeeper

Another strategy to block the pores is by the direct electrostatic interaction between the surface of the nanoparticles and the gatekeeper. Essentially, the mechanism is the same as in the previous section, but here, there is only one type of polyelectrolyte, and the surface has to be functionalized according to that. Then, when the pH changes, the electrostatic interactions between the gate and the nanocarrier decrease, and the pores are opened.

Although MSNs capped with carbon dots through electrostatic interactions have been reported [153], the most extended procedure is to use a polyelectrolyte polymer to block the pores, as was first proposed back in 2005 [154]. Chitosan can be directly adsorbed by hydrogen bonding onto the surface to form a single protective layer. When pH drops, the amine groups get protonated, and the polymer shell swells reversely, allowing the release and making it a good on/off pH-responsive system [140,141,142,143]. The covalent bonding of chitosan onto the surface through cross-linking chemistry has also been reported [155,156,157,158].

The use of polyethyleneimine (PEI) conjugated with folic acid has also been reported [147,148]. This strategy combines an improved cellular uptake due to the interactions between folic acid and overexpressed folate receptors in some cancer cells with an efficient electrostatic interaction between positively-charged PEI and negatively-charged nanoparticles. Other polycations, such as polyvinyl pyridine [149] or poly(2-diethylamino ethyl methacrylate) [159], have been reported, as well as polyanions, such as poly(acrylic acid-co-itaconic acid), in combination with human serum albumin to enhance its biocompatibility [150].

The use of gelatins electrostatically adsorbed onto the surface of MSNs as pH-responsive nanogates has recently been explored by our group [160]. The gelatin presents ionizable groups that interact with the surface. In acidic medium, the swelling of the gelatin is mainly controlled by the protonated amine groups, while in basic medium, that is controlled by the deprotonated carboxylic acid groups. This behavior leads to a great release of the water-soluble drug topotecan only at acid pH, where the drug is in its active form.

#### 4.2.3. Cyclodextrins

Cyclodextrins (CD) are water-soluble and biocompatible macromolecules obtained from the enzymatic degradation of starch. They have a hydrophilic outer surface and a hydrophobic cavity that allows a number of structures to complex inside. Among all of the existent CD, the preferred ones in drug delivery are β-CD due to the perfect cavity size and complexation ability [161]. Although β-CD covalently attached to the surface have been reported [162,163], β-CD-based drug delivery systems are usually composed of a covalently-linked amine-based stalk attached to the surface of the nanoparticles that is capable of interact electrostatically with the hydrophobic cavity of β-CD. At neutral pH, the stalk and the cap interact closely, while at lower pH, the affinity between the stalk and the cap decreases, thus opening the pores and leading to systems with high efficacy in the treatment of cancer [164] or bacterial diseases [165]. Interestingly, β-CD gates whose on/off behavior is not driven by the mentioned general mechanism (positive amine complexed into the negative cavity) have been reported. Such modification could be achieved by modifying the stalk and the β-CD with complementary base pairs [166].

### 4.3. Gatekeepers Suffering Conformational Changes

**Figure 7:**
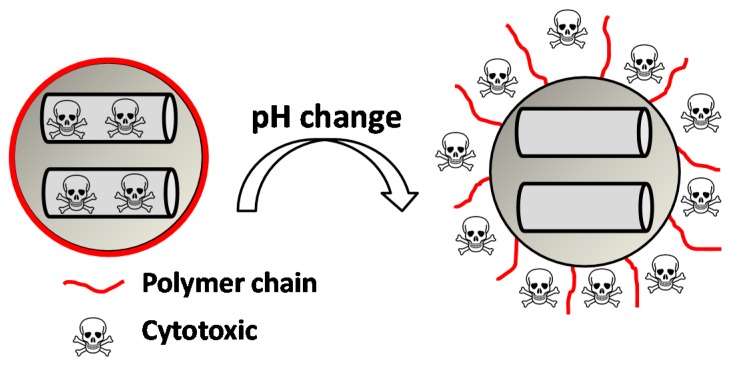
Collapsed-to-extended phase transition of a polymer upon changes in pH triggering the cytotoxic release.

So far, we have described some pH-responsive gatekeepers containing various types of organic moieties capable of avoiding premature release by the electrostatic interaction with other polymers or being part of other pH-responsive mechanism among others. However, from now on, we will focus on supramolecular structures that can suffer conformational changes themselves under pH changes. The behavior of these gatekeepers, mainly polymers, is quite simple. Generally speaking, at neutral pH, the gate is in a collapsed state on the surface, thus blocking the pores; then, when the pH changes to acidic or basic pH, depending on the nature of the gate, the gate opens in some way, and the drug is released (Figure 7).

This behavior is based on the presence of anionic or cationic functional groups that remain neutral at physiological pH, but acquire net charge under pH changes, thus leading to repulsion forces between the chains and to the change from hydrophobic to hydrophilic. The advantage of this is that by choosing the convenient functional group, it is possible to design a system with a concrete pKa suitable for a specific disease. Some of the polymers that behave in that way are shown in Table 3.

#### 4.3.1. Anionic Polymers

Anionic polymers are usually composed of monomers that contain the carboxyl functional group, although sulfonic-based polymers have been reported [191]. These polymers remain neutral below certain acidic pH values, depending on the pKa of the polymer, but become negatively charged at higher pH. Then, their expected behavior is to be collapsed on the surface when the pH becomes acid, avoiding premature release, and to expand when pH rises again, thus allowing the drug release. Poly(acrylic acid) (PAA) has pKa = 3, and it has been shown that at pH > 4, the carboxylic acid groups become protonated. Then, this gate can avoid the release at very acidic pHs, which would constitute it as a promising gatekeeper for oral drug delivery, where the stomach juices have to be avoided [167,168,169]. However, this polymer has also been applied to acid-targeted drug delivery, as it has been shown that DOX can interact electrostatically with the PAA shell at neutral pH, while when the pH drops, the interactions weaken, and DOX is released [170,171,172]. Poly(methacrylic acid) (PMAA) has pKa = 5–6, and as PAA, it has been applied to base-targeted drug delivery by releasing ibuprofen [173,174], as well as to acid-targeted drug delivery by releasing DOX [175]. PMAA has been copolymerized with methyl methacrylate (commercial name: Eudragit S-100) and applied to oral drug delivery [176,177]. PMAA has been widely applied to the synthesis of multi-responsive drug delivery systems. Although the most extended strategy is the copolymerization of PMAA with poly(*N*-isopropylacrylamide) (PNIPAM) to yield thermo- and pH-responsive nanocarriers, PMAA copolymerized with poly(*N*-vinylcaprolactam) (PVLC) and cross-linked through disulfide bonds to give a thermo-, redox- and pH-responsive drug delivery system has also been reported [178], as well as a thermo-, light- and pH-responsive nanocarrier by using up-converting nanoparticles [179,180]. The addition of PMAA to temperature-responsive PNIPAM has been shown to modify the temperature at which the volume phase transition takes places, i.e., the temperature at which the pores are opened. The more PMAA, the higher the temperature is necessary for the transition to occur. Moreover, as at neutral pH, PMAA is hydrophilic, it can reduce the plasma proteins’ adsorption [192,193,194]. The copolymerization of PMAA and PNIPAM with soy phosphatidylcholine, a phospholipid, to increase biological stability has also been reported [195].

#### 4.3.2. Cationic Polymers

Cationic polymers are composed of aminated monomers that remain neutral at physiological pH and become protonated when the pH drops below a certain value. Then, this gate avoids the release until the nanocarrier is internalized by the cell, where the acidic organelles protonate the amine groups of the polymer chain, thus opening the pores. Multiamine chains have been used as gatekeepers [181], but the use of more complex and functional structures is common. Poly(L-histidine) has been applied to MSN as a cationic gatekeeper due to the pKa of its imidazole ring [196]. Poly(vinyl pyridine)-based polymers have been reported. Poly(4-vynil pyridine) polymers provide nanocarriers with pH-responsive functionality [197,198], but they have a relatively low full-protonation pH, which makes poly(2-vynil pyridine) polymers more suitable to be applied to cancer tissues [199]. The use of pH-responsive polyamidoamine (PAMAM) dendrimers has also been reported [182]; however, the use of pentaethylenehexamines as pH-responsive end groups for dendrimers seems preferable, since PAMAM-based nanocarriers show cytotoxicity when exceeding the third generation [183]. Interestingly, the combination of acid-labile bonds that lead to a cationic polymer after bond hydrolysis has been reported [200]. Moreover, the combination of imine-based pH-detachable PEG with a polymer that suffers charge reversal from negative to positive as a coating has also been reported [201].

In the previous section, we described PAA and PMAA acting alone or copolymerized with other sensitive polymers. However, acrylates and methacrylates can be used as part of the backbone of amine-based monomers that can lead to polymers capable of suffering a hydrophobic to hydrophilic phase transition when the pH drops below physiological pH due to the protonation of its tertiary amine groups. Poly(2-(dimethylamino)ethyl acrylate) (PDMAEA) has been used as a pH-responsive gatekeeper alone [184] or combined with light-responsive moieties [185]. Recently, poly(2-(pentamethyleneimino)ethyl methacrylate) (PPEMA) has been proposed as a pH-sensitive gate [186], although the most used amine-based methacrylate is poly(2-(diethylamino)ethyl methacrylate) (PDEAEMA). The use of PDEAEMA as a gate itself has been reported [187,188], as well as being part of multi-responsive drug delivery systems. It has been copolymerized with PNIPAM to give a thermo- and pH-responsive polymer [189]. Moreover, it has been used in combination with redox- and light-responsive bonds to give rise to a nanocarrier with enhanced killing capacity [190].

#### 4.3.3. Peptides and More Complex Biomolecules

It is well known that peptides, proteins and DNA can suffer conformational changes when exposed to heat or changes in pH, thereby going from a more packaged structure to a lesser one. This behavior can be used in the design of on/off pH-responsive gates, as these phase transitions are known to be reversible. These structures can be used in the design of nanocarriers for oral drug delivery where resistance to acid pH is needed. DNA capable of suffering a phase transition from quadruplex to single strand when the pH increases from acid to neutral pH has been reported [202], as well as lysozyme proteins that can block the pore when the pH drops and allow the drug release when recovering neutral pH [203]. However, the design of biomolecule-based nanocarriers that allow the release when the pH changes to acidic values is more common. DNA can be used as a reversible linker between small gold nanoparticles and MSNs [204]. Adenine DNA has been used as a capping agent taking advantage of the instability of the non-Watson–Crick secondary structures when the pH drops [205]. Double DNA strands formed by the interaction of thymine bases with Hg^2+^ ions that suffer the phase transition to single strand when the pH drops have also been reported [206], as well as small peptides that suffer the β-sheet-to-random coil transition [207].

## 5. Conclusions

The state-of-the-art of pH-responsive drug delivery using mesoporous nanoparticles has been deeply revised. These stimuli-responsive nanocarriers are based on the small variations in pH between healthy and diseased areas along the body. Those variations are used to trigger the release of the cargo through different mechanisms. We have considered three different approximations of pH-responsive nanocarriers. The host-guest interactions have been shown to be useful to retain the drugs at physiological pH. However, it would be desirable to minimize the amount of drugs released outside the target area despite the electrostatic interactions. To achieve that effect, a number of gatekeepers have been developed to block the pores, either via acid-labile bonds or pH-responsive supramolecular structures (polymers, nanoparticles, etc.), thus leading to systems with proven efficacy in vitro and in vivo.

Regarding the design of the mesoporous nanocarriers, the future work should be directed toward: (1) scaling up the synthesis of those smart nanocarriers while minimizing the expenses; (2) obtaining a complete characterization of the nanocarriers from both the physico-chemical and toxicological point of view; (3) carrying out accurate studies of the biodistribution in humans; and (4) determining the real possibilities of nanoparticle-based therapies against diseases, such as cancer. Regarding the pH-responsive nanocarriers, it has been shown in this review that it is possible to design hybrid nanoparticles capable of triggering the release in very specific situations through the subtle changes in pH. However, it is still difficult for all of the nanocarriers injected to reach the diseased area. Then, future work should be directed toward synthesizing smart pH-responsive nanocarriers with enhanced targeting ability, capable of avoiding the different barriers present in the body and accumulating only in the diseased tissues to increase the efficacy of the therapy.

## Figures and Tables

**Table 1:** Acid-labile bonds that have been employed for the design of different pH-responsive mesoporous nanoparticles.

pH-Responsive Bond	pH-Responsive Mechanism	References
Hydrazone	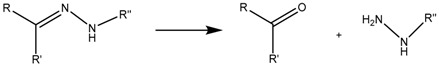	[70,71,72,73,74,75,76,77]
Acetal	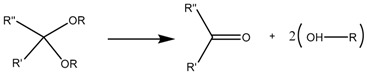	[78,79,80,81,82]
Imine	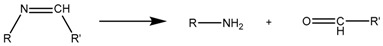	[83,84,85,86,87,88,89]
Ester-based	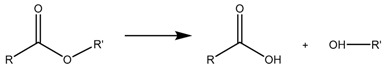	[90,91,92,93,94,95,96,97,98,99]
Citraconic	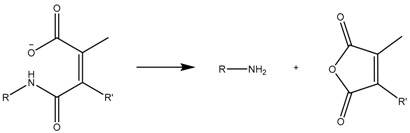	[100,101]

**Table 2:** Polyelectrolytes most commonly used in the design of pH-responsive gates through electrostatic interactions between them or with the nanocarrier.

Polycation	Structure	References
Chitosan	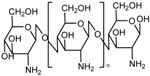	[140,141,142,143]
Poly(allylamine hydrochloride)	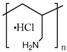	[144,145,146]
Polyethyleneimine	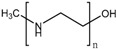	[147,148]
Polyvinyl pyridine	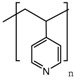	[149]
Polyanion	Structure	References
Poly(styrene sulfonate)	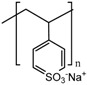	[146]
Poly(acrylic acid-co-itaconic acid)	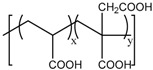	[150]

**Table 3:** Phase-transition polymers usually employed as gatekeepers in pH-responsive nanocarriers.

Anionic Polymers	Structure	References
PAA	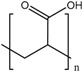	[167,168,169,170,171,172]
PMAA	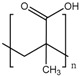	[173,174,175,176,177,178,179,180]
Cationic Polymers	Structure	References
Polyamine-based	--	[181,182,183]
PDMAEA	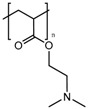	[184,185]
PPEMA	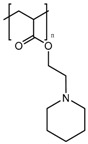	[186]
PDEAEMA	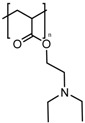	[187,188,189,190]

PAA: poly(acrylic acid); PMAA: poly(methacrylic acid); PDMAEA: poly(2-(dimethylamino)ethyl acrylate); PPEMA: poly(2-(pentamethyleneimino)ethyl methacrylate); PDEAEMA: poly(2-(diethylamino)ethyl methacrylate).
